# Male Dispersal Pattern in Golden Snub-nosed Monkey (*Rhinopithecus roxellana*) in Qinling Mountains and its Conservation Implication

**DOI:** 10.1038/srep46217

**Published:** 2017-05-11

**Authors:** Zhi-Pang Huang, Kun Bian, Yi Liu, Ru-Liang Pan, Xiao-Guang Qi, Bao-Guo Li

**Affiliations:** 1 College of Life Sciences, Northwest University, Xi’an, Shaanxi 710069, China; 2 Shaanxi Key Laboratory for Animal Conservation, Northwest University, Xi’an, Shaanxi 710069, China; 3 Institute of Eastern-Himalaya Biodiversity Research, Dali University, Dali, Yunnan 671003, China; 4 School of Anatomy, Physiology and Human Biology, The University of Western Australia 6009, Perth, Western Australia, Australia

## Abstract

Golden snub-nosed monkey (*Rhinopithecus roxellana*) is one of the most endangered primate species found in China, exhibiting multilevel society consisting of several one-male-females together with their offspring units (OMU), and all-male units (AMU). Female dispersal patterns of the species within herd have been well documented, whereas those of the males within or between herds are still poorly understood. Our results based a long-term observation indicate that more than half of sub-adult males, and half of the deposed males that stayed a short period in OMU disperse between herds, three of them established their own OMU in new herd after the dispersal. Smaller number of the sub-adult and adult males, compared with adult females, stayed in natal herd, implying sub-adult males started dispersing and male-biased dispersal occurred between herds. High frequencies of resident males were wounded as their OUMs were taken over, and resident males co-operation defend bachelor males were found. Mating competition among males within the herd may have contributed to the scenarios of male-biased dispersal. The results also suggest that maintaining connection between isolated herds and establishing the corridors among the fragmented habitats for the species will greatly benefit increasing its gene flow and promoting conservation status.

The diversity of animal social systems represents an evolutionary alternative developments and adaptations responding to environmental and social pressures^1^,^2^,^3^. Many studies have focused on the evolution of social system in primates and their selective pressure, which have provided supplementary information and references in understanding the same issues on human beings^3^,^4^,^5^, but less effort has been devoted to the understanding of how primates’ social systems show adaptive behaviors when their envionments have rapidly changed^6^. Nevertheless, such understanding is critical to developing effective conservation strategies, policies and managements for protecting endangered primates like the golden snub-nosed monkey (*Rhinopithecus roxellana*).

Dispersal behaviors are the primary strategies and necessities for maintaining the connection between subgroups and groups, which create diversities in many aspects during evolution^7^,^8^. With regard to dispersal patterns sex-biased dispersal (SBD), such as male-biased one (MBD), is very common in mammals, and female-biased one (FBD) has also been found in some animals, for instance birds^9^. As for nonhuman primates, three different kinds of dispersal patterns - male-biased, female-biased and bisexual dispersal - have been reported^4^,^8^,^10^,^11^,^12^, and mating system is regarded to affect the patterns that results to SBD^9^. Höner^13^ points out that the evolution of male-based dispersal patterns is supposed to be driven by female mate preferences, depending on demographic structure of breeding groups, rather than genetic relatedness between sexes.

Moreover, dispersal rate is also impacted by environmental change and habitat disturbance. For example, the increasing dispersal rates of both male and female Japanese macaques (*Macaca fuscata*) are considered to be associated with the decreasing of the provisioned food^14^,^15^. On the other hand, deforestation and subsequent habitat fragmentation made significant contribution to the high dispersal rate in female mantled howlers (*Alouatta palliata*) in Costa Rican tropical dry forest^16^.

The golden snub-nosed monkey studied is one of the most endangered species of Asian colobines and endemic to China. Its distribution is restricted to temperate montane forests at 1,000–4,100 m above sea level across three isolated regions (i.e., Sichuan and Gansu, Shaanxi, and Hubei provinces) in central and southwestern China^17^,^18^,^19^. Unlike other colobines with small group size in harem and small home overlapping range, *R. roxellana* lives in large groups, varying from 100 to 400 individuals^20^,^21^,^22^. Its social organization is described as a multilevel or modular society, composing several independent harem or one-male breeding units (OMU) that consists of a single resident adult male, adult and sub-adult females, juveniles and infants^23^,^24^,^25^,^26^. Multiple OMUs and one to three of all male units (AMUs, non-breeding units) travel, forage, feed, and rest together to form herd, two or more herds may temporarily merge to form a single large troop^4^,^18^,^19^,^27^.

Several studies have been carried out on dispersal patterns of the species from both behavioral and genetic points of view. With regard to the definition of male disperse, those who have not been observed again after births in natal herd were considered to have dispersed to other neighboring herds^24^,^28^. The male and female dispersals of the species have been identified according to the d-loop region of the mtDNA in Qinling Mountains^29^. Its male dispersal patterns in Shennongjia have also been reported with polymorphic microsatellite^30^. Some of the hypotheses made previously are, however, needed to be clarified with more robust evidence, particularly referring to the scenarios found in other regions, such as the region studied in this project, since the dispersal pattern is remarkably environmentally related. And it is critical to see such diversity. On the other hand, there has no study monitoring the life history of males before and after their migration. For example, although behavioral response of the deposed male to his former OMU females in natal herd has been studied^30^, the whole life history of the males emigrated into neighboring herds has not been traced.

Thus, the main purposes of this study include: (1) monitoring life history of the male golden snub-nosed monkeys before and after their disperse; (2) describing sex ratio differentiation of the alternative sex-age groups in a semi-provisioned herd, then exploring dispersal pattern of the species with hierarchical social structure; and (3) assessing the factors causing male-biased dispersal.

## Results

### Dispersal pattern

Seventeen sub-adult males were tracked, 11 of them (64.7%) leaved their natal herd before they got sexually matured, and the rest of them stayed in the herd during the study period. Eight of the eleven sub-adult males disappeared, the other three were found in neighboring herd, and one of them had established his own OMU in the new herd. All of the six sub-adult males stayed in natal herd finally established their OMU, while one of them lost his OMU’s term in one year.

Twelve resident males established their OMU that was taken over by other males during the study period. Half of the deposed males who’s leadership of OMU lasted for 8 years on average (from 4–12 years, *n* = 6) still stayed in natal herd; one of them established his OMU with two former females for 3 months. Another six deposed males maintained their leadership of OMU for less than one year (from about 3 to 8 months); three of them still stayed in natal herd, and rest of them emigrated to the other herds. One of the three deposed males established his OMU in their natal herd. Similarly, one of the three emigrated males was found in DJF herd and has established his OMU; the rest two had dispersed to other herds.

Twenty adult females were monitored during the period; only four of them disappeared for unknown reason, and other 16 stayed in natal herd. Three of them unfortunately died in the following two years.

### Male dispersal between herds

**Figure 1:**
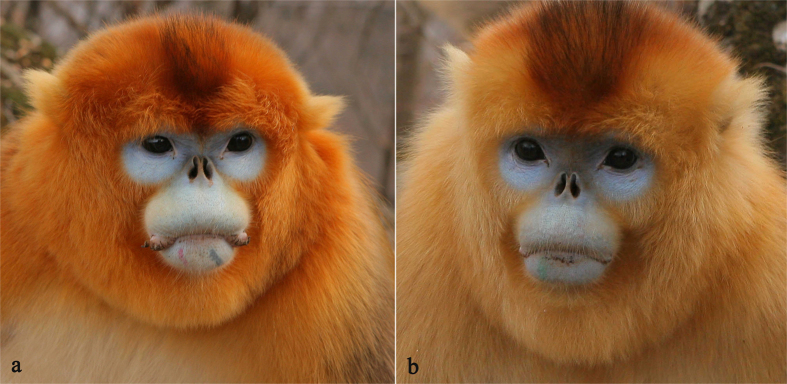
The two male golden snub-nosed monkeys emigrated from their natal herd and established harems in neighboring herd in Qinling Mountains. BZ (**a**) with red and black tattoo in the lower lip, and R_1_G_3_ (**b**) with green tattoo in the lower lip.

Ten males (3 adults and 7 sub-adults) from GNG herd were found to follow DJF herd in Dec. 2012. Two of them were identified due to the tattoos in their lips; one was a sub-adult male R_1_G_3_, and the other was BZ who was a resident male replaced three months ago before getting out GNG herd (Fig. 1). One adult male, BeiDou (BD), which had established his OMU in DJF herd, has well habituated to observers, indicating it came from GNG herd. R_1_G_3_ and BZ were found to be resident males of OMUs in DJF herd in Dec. 2014. OMU of R_1_G_3_ had two adult females, one sub-adult female and two juveniles (about 2 years old) and one nearly yearling infant. OMU of BZ had only one adult female and one nearly yearling infant. Moreover, no any male was found to emigrate from GNG herd with R_1_G_3_, and BZ stayed in DJF herd.

### Number of male and female in GNG herd

Both breeding band and herd included more females than males in GNG herd. There was no significant difference in sex ratio in the aged groups, from infant to three years old juniors, in GNG breeding band (Table 1). More females than males stayed in GNG-breeding band (18 vs. 56, *Z* = 5.15, *P* < 0.001), resulting in significant sex ratio differentiation in the GNG-breeding band (56 vs. 112, *Z* = 4.58, *P* < 0.001, Table 1). No sex ratio differentiation was found in the aged groups, from infant to four years old juniors, in GNG herd, which is significantly different from that between males, including sub-adult males and adult males, and adult females (39 vs. 56, *Z* = 1.77, *P* < 0.038), and more females were found in GNG herd (*Z* = 1.71, *P* < 0.043, Table 1).

### Mating competition among males

Thirteen events of OMU taken over were detected, six of resident males were serious wounded from new males during fighting. Two of them, BB and HB, were heavy wounded in hind legs. A total of 69 instances of resident males collectively attacked bachelor males in AMU were recorded, which is at least three resident males co-operation, between Oct. 2012 and May 2016. A highly ranked adult male B_4_ in AMU was severely injured in his hind legs by resident males. In another cases, two males whose OMU had been taken over five months ago was severely wounded resident males while it tried to re-establish his OMU in GNG herd, and one of them was died in one week.

## Discussion

Our study first described dispersal patterns of the golden snub-nosed monkeys between herds based on a long-term observation and individual identification and life history monitoring. Given the dispersal pattern between herds in the species is based on the phenomenon of male disappearing in natal herd and the corresponding support from genetic analysis^24^,^25^,^29^,^31^, we found three males successfully established their OMU in a new herd, and two of them did the same thing after two years of leaving. We also found that juvenile males emigrated from their natal OMU at the age of 3–4 years old, and male secondary disperse between herds mainly occurred in sub-adults. It has been reported that sex-biased mortality occur in some primate species: males with higher death rate than females^32^, which may sometimes cause confusion with sex ratio differentiation at birth. Owing to no significant sex ratio difference from infants to four years old juveniles was found, the differentiation in sex ratio of GNG herd can be considered due to the unbalanced sex number of sub-adult and adult males, and the staying of adult females in the herd; only one resident male and three older adult females were reported dead during the study period. That twenty percent of adult females was found to disperse between herds in this study is similar to the reports from previous studies^29^. Therefore, the number differences between sub-adult and adult males, and adult females found in our study imply that male dispersal occurred between the herds.

Mating system in multilevel society of some non-colobine primates is more complicated than that of the colobines, and its impacts on dispersal pattern are varied, such as in gelada (*Theropithecus gelada*) mating behaviors are excluded both follow male and other OMU resident males, and it exhibits male-biased dispersal which results to more females in OMU within herd^33^. As for hamadrayas baboon (*Papio hamadryas*) mating behaviors are also limited to OMU males, showing a female-biased dispersal within band^34^,^35^. This indicates that the impacts of mating system on dispersal pattern may be affected by other ecological factors. With regard to the golden snub-nosed monkeys, its mating behavior is not limited to resident male of OMU, other resident males and bachelors also have chances to mate with OMU females^26^,^27^,^36^,^37^. As a result, both males and females were found to disperse within herd^24^.Even though we found some healthy females to leave their natal herd, their dispersal frequency was lower than that reported from a previous study based on genetic method^29^. This is similar to the patterns observed on the populations in Shennongjia^31^.

Society characterized with polygyny or operating sex ratio in OMU is a scale predicting male mate competition^5^,^38^, which may favor for males’ dispersal between herds. With regard to the monkeys studied, only half adult males stayed in GNG herds were leader males, and on average there are four adult females in each OMU^24^, implying high male mating competition in the herds. We observed several injury cases among bachelors or resident males during the formation of a new OMU, and a high frequency of resident males collectively attacked bachelors who were close to breeding band was found in GNG herd, as occurred in Shennongjia^39^. Fourteen resident males collectively attacked one bachelor in GNG herd, which resulted in more wounded bachelors in AMU. All of these imply that male mating competition within herd may be one of the main factors driving male golden snub-nosed monkey’s dispersal between herds, especially sub-adult males and the deposed resident males.

The previous report indicated that female mating choice and inbreeding avoidance also play an important role in the formation of a new OMU, triggering females to prefer to young residents and immigrated males^24^. It is interesting that BZ was not injured during the taking over of his OMU. He dispersed together with other males in natal herd and successfully established a new OMU in JDF herd. Thus, we hypothesize that female mate choice partly makes contribution to male dispersal between the herds in *R. roxellana* in Qinling Mountains.

The results found from this study indicate that the monitoring of dispersal patterns of the species, particularly endangered ones, is very critical in understanding dynamic social profiles of the species, and developing effective conservation strategies and measures to increase gene flow and reduce the risk of inbreeding depression^40^,^41^. Distribution areas of the snub-nosed monkeys have sharply shrunk in the past 400 years due to rapid growing human populations and serious deforestation, particularly since the second half of the last century^12^. Subsequently this has resulted in a serious problem of gene follow and the fragmented herds. Therefore, male dispersal and successfully establishing new OMU in new herd is the way of overcoming such problems. In other words, habitat connection among populations and between herds for the species will be a great benefit for their conservation and development, particularly regarding the populations in the three major isolated distribution area- Shennongjia, Qinling Mountains and Wolong nature reserve^4^,^18^,^19^,^42^. Our results have also provided very valuable information and evidence for improving primate conservation, especially the other species of the snub-nosed monkeys, for example, the black-and-white species (*R. bieti*) in Yunnan and Tibet, which has been found that half of its existing populations are isolated one from another, and three of them are on the way of extinction^43^. Thus, our study further highlights: habitat recovery and corridor establishment between the populations of the *Rhinopithecus* are extremely critical.

## Methods

### Study site and subjects

The project was carried out at Yuhuangmiao, Zhouzhi National Nature Reserve on the northern slope of Qinling Mountains in Shaanxi, China (108°14′–108°18′E, 33°45′–33°50′N), with an altitude from 1,400 to 2,890 m a. s. l., and an average annual temperature of 10.7 °C (maximum 31.5 °C in July and a minimum −14.3 °C in January), and average annual rainfall is 894 mm^25^. The vegetation of the area consists of deciduous broadleaf forest (1,400–2,200 m), coniferous and deciduous broadleaf mixed forest (2,200–2,600 m) and coniferous forest (above 2,600 m)^44^. The terrain is extremely mountainous with considerable seasonal variation in ecology and major food availability of the species studied^45^.

**Figure 2:**
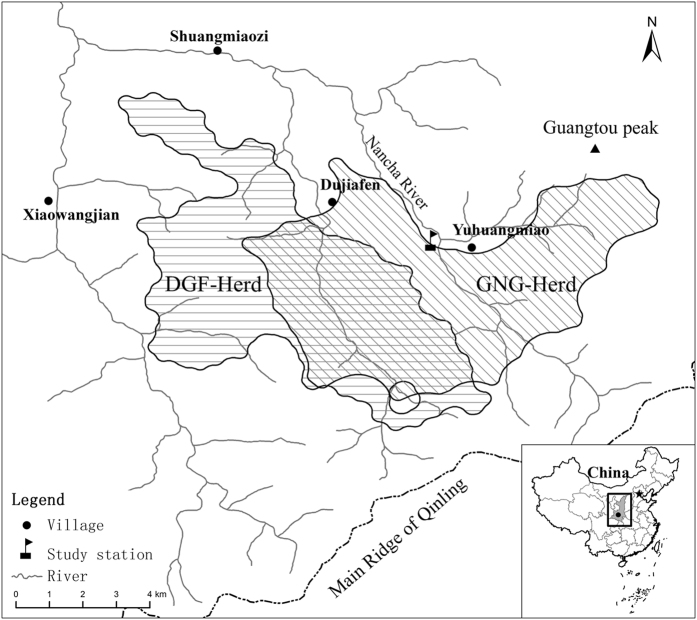
Study site and home range of the two golden snub-nosed monkey herds in Qinling Mountains. Data of home range of study herd obtain from position of GIS collar carried by the monkeys^4^; home range area of two focus herds was made by positions with 200 m buffer radius using QGis^48^.

Two troops of the monkeys (ERT: East Ridge Troop, and WRT: West Ridge troop) live in the area^44^, sharing overlapping home ranges^42^, and separated by Nancha river (Fig. 2). Our study focused on the WRT troop that predominantly inhabits the West Ridge. Several studies on the behavior ecology and conservation of the troop have been carried out from October 2001, 6–8 months/year^24^,^25^,^46^,^47^. The WRT troop split into two herds in 2002, one is the GNG living around the semi-provisioned site, and has been well habituated to observers. The other is DJF, the west of GNG (Fig. 2), a wild free ranging herd without provision, and it was initiated to be habituated by provisioned in Dec. 2012. The two herds exhibit occasional fusions in winter, and there were some new individuals or disappeared ones in the GNG herd, indicating emigration or immigration occurs frequently^4^.

Having GNG herd members become more accustomed to the presence of the observers, studies on behaviors were carried out at a distance about 20 m. Individual identity was based on body characters, such as pelage coloration, crown hair pattern, scars or evidence of previous injury, and other prominent physical features, such as the shapes of granulomatous flanges on both sides of the upper lip^24^,^25^ and the tattoos with unique individual sequence of numbers and colors (red, black, green and blue) on the upper or lower lips^4^. Sex-aged categories were defined in this way: adult males (more than 7 years old), sub-adult males (4–7), adult females (more than 4) who would give first birth, sub-adult females (3–4), juveniles (1–3), and infants (less than 1).

Social organization of the studied herds included two levels: breeding band and all-male groups. There were 14 OMUs in the breeding band, each with one adult male and 47 adult and 12 sub-adult females in GNG herd. The average number of adult and sub-adult females in each OMU was 4.2 ± 1.6 (ranging from 2–7). There were 32 individuals in all-male group that fed and moved together with the breeding group including 18 adults, 13 sub-adults and 11 juvenile males in Jan. 2015, respectively. With regard to the DJF herd, it consists of 7 OMUs and all male group (10 sub-adult and adult males, and 2 juvenile males), totally 70.

### Data collection and statistics

We counted all of OMUs one by one in breeding band, recoded the numbers of adult females, sub-adult females, juveniles and newborn infants. We also identified sex of all juveniles and newborn infants. Dispersal events of sub-adult males, resident adult males and females were monitored from Dec. 2009 to May 2016 for GNG herd, including two periods, Oct. to Jan. and Mar. to May every year. Seventeen sub-adult males, twelve deposed male and twenty females, who emigrated from natal herd, were recorded to clarify whether they combined with new herd. For evaluating the extent of mating competition among males, we also recorded the wound on resident male during his OMU was taking over, and bachelor males in AMU after resident males collectively attacked^39^, within a total 1,917 hours of observation in GNG herd.

Proportion test was used to evaluate sex ratio differentiation of the all age-categories of both GNG herd and its breeding band. The significant level was set at *P* < 0.05.

### Ethics statements

Research protocols for the study was granted by the Chinese Academy of Science, complied with the principles approved by animal care committees of the Wildlife Protection Society of Shaanxi Province, China, and adhered to the regulatory requirements of Zhouzhi National Reserve, China, and to the American Society of Primatologists principles for the ethical treatment of primates.

## Additional Information

**How to cite this article**: Huang, Z.-P. *et al*. Male Dispersal Pattern in Golden Snub-nosed Monkey (*Rhinopithecus roxellana*) in Qinling Mountains and its Conservation Implication. *Sci. Rep.* **7**, 46217; doi: 10.1038/srep46217 (2017).

**Publisher's note:** Springer Nature remains neutral with regard to jurisdictional claims in published maps and institutional affiliations.

## Figures and Tables

**Table 1:** Secondary sex ratio of the GNG breeding band and herd of the golden snub-nosed monkeys in Qinling Mountains.

Social components	Sex	Adult	J_4_	J_3_	J_2_	J_1_	I	Total
GNG-breeding band	Male^a^	18	0	5	9	13	11	56
	Female	56	9	11	13	10	13	112
	Z*	5.15		0.85	0.75	0.73	0.41	4.58
	p	**0.001**		0.2	0.23	0.23	0.34	**0.001**
All male band	Male	21	4	4	3	0	0	32
GNG-Herd (Total)	Male	39	4	9	12	13	11	88
	Female	56	9	11	13	10	13	112
	Z	1.77	0.6	0.05	0.2	0.73	0.41	1.71
	p	**0.038**	0.28	0.48	0.42	0.23	0.34	**0.043**

^a^GNG breeding band consists of 15 OMUs, with 15 and 3 sub- adult males staying in natal OMUs; J_4_, juvenile aged 4; J_3_, juvenile aged 3; J_2_, juvenile aged 2; J_1_, yearling infant and I, newborn infant. *proportion test.
